# Differential seeding and propagating efficiency of α-synuclein strains generated in different conditions

**DOI:** 10.1186/s40035-021-00242-5

**Published:** 2021-06-21

**Authors:** Di Liu, Jian-Jun Guo, Ji-Hui Su, Alexander Svanbergsson, Lin Yuan, Caroline Haikal, Wen Li, Gunnar Gouras, Jia-Yi Li

**Affiliations:** 1 Institute of Neuroscience, College of Life and Health Sciences, Northeastern University, Shenyang, 110169 China; 2 Neural Plasticity and Repair Unit, Wallenberg Neuroscience Center, Lund University, BMC A10, 22184 Lund, Sweden; 3 Experimental Dementia Research, Lund University, BMC B11, 22184 Lund, Sweden; 4 Institute of Health Sciences, China Medical University, Shenyang, 110122 China

**Keywords:** α-Synuclein, Strains, Prion-like propagation, Parkinson’s disease

## Abstract

**Background:**

Accumulation of alpha-synuclein (α-syn) is a main pathological hallmark of Parkinson’s and related diseases, which are collectively known as synucleinopathies. Growing evidence has supported that the same protein can induce remarkably distinct pathological progresses and disease phenotypes, suggesting the existence of strain difference among α-syn fibrils. Previous studies have shown that α-syn pathology can propagate from the peripheral nervous system (PNS) to the central nervous system (CNS) in a “prion-like” manner. However, the difference of the propagation potency from the periphery to CNS among different α-syn strains remains unknown and the effect of different generation processes of these strains on the potency of seeding and propagation remains to be revealed in more detail.

**Methods:**

Three strains of preformed α-syn fibrils (PFFs) were generated in different buffer conditions which varied in pH and ionic concentrations. The α-syn PFFs were intramuscularly (IM) injected into a novel bacterial artificial chromosome (BAC) transgenic mouse line that expresses wild-type human α-syn, and the efficiency of seeding and propagation of these PFFs from the PNS to the CNS was evaluated.

**Results:**

The three strains of α-syn PFFs triggered distinct propagation patterns. The fibrils generated in mildly acidic buffer led to the most severe α-syn pathology, degeneration of motor neurons and microgliosis in the spinal cord.

**Conclusions:**

The different α-syn conformers generated in different conditions exhibited strain-specific pathology and propagation patterns from the periphery to the CNS, which further supports the view that α-syn strains may be responsible for the heterogeneity of pathological features and disease progresses among synucleinopathies.

**Supplementary Information:**

The online version contains supplementary material available at 10.1186/s40035-021-00242-5.

## Background

Accumulation of misfolded α-synuclein (α-syn) is associated with a group of neurodegenerative diseases, collectively known as synucleinopathies, including Parkinson’s disease (PD), dementia with Lewy bodies (DLB), multiple system atrophy (MSA) and pure autonomic failure [1–4]. α-Syn aggregates are found in neurons as Lewy bodies (LBs) and Lewy neurites (LNs) in PD and DLB [5, 6]. In MSA, α-syn mainly accumulates and forms glial cytoplasmic inclusions [7] in oligodendrocytes. Synucleinopathies are clinically and pathologically heterogeneous [8], as different regions of the central nervous system (CNS) are affected in distinct diseases [9–12], suggesting the heterogeneity among distinct α-syn strains.

α-Syn is a 140-amino-acid long cytoplasmic protein enriched in presynaptic nerve terminals [13, 14]. Native α-syn is unfolded and soluble, and adopts an α-helical conformation upon binding to the membrane. However, under pathological conditions, it aggregates into oligomers and fibrils and forms insoluble β-sheet-rich amyloid structures [6]. The misfolded α-syn exhibits a “prion-like” self-propagation manner in the neuronal network. The α-syn can self-propagate and progressively spread between interconnected brain regions *via* cell-to-cell transmission mechanisms. In addition, evidence supporting this is that the pathological α-syn accumulation has been shown in different brain regions in PD patients, following a caudo-rostral pattern [10, 15–17]. Moreover, embryonic mesencephalic neurons transplanted into the striatum of PD patients developed LB pathology more than 10 years after transplantation, suggesting the host-to-graft LB pathology in the PD patient brains [18–20].

Previous studies have shown that brain homogenates from patients with synucleinopathies and transgenic animals could induce varied α-syn pathologies in cellular and animal models [12, 21–26]. α-Syn preformed fibrils (PFFs) generated from purified recombinant α-syn behaved similarly in morphology, biophysics, and biochemistry as those extract from the brain homogenates of the patients [27]. Multiple studies have shown that PFFs can act as seeds and further induce α-syn pathology both in vitro and in vivo [4, 21, 27–34]. More recently, studies have suggested that α-syn strains have distinct structural and biochemical characteristics, and these fibrils behave as strains, with different seeding capacities [35–38]. Taken together, the conformational α-syn polymorphs are thought to contribute to different lesions in PD and related diseases. However, there is a lack of direct comparison of the seeding potency and propagation from the periphery to the CNS among different α-syn conformers. Moreover, it remains unclear how different α-syn strains induce different pathologies, and the factors influencing the seeding and spreading process of α-syn from the periphery to the CNS still need further elaboration, from both views of endogenous cellular environment [39, 40] and the strain-specific characteristics of α-syn aggregates.

In this study, we set out to address these questions by employing three different α-syn PFFs generated under different conditions. We proposed that different α-syn strains display distinct seeding effects and propagation propensities from the peripheral nervous system (PNS) to CNS. Since intramuscular (IM) injection of α-syn can induce pathology in the CNS [41], we injected the three α-syn strains into the skeletal muscle of a Bacterial Artificial Chromosome (BAC) transgenic mouse expressing wild-type human α-syn, and examined the pathological features at 180 days post injection (dpi). Notably, we observed strain-dependent seeding and propagating pathology throughout the spinal cord and the brain to different extents. Our results suggest that different strains of recombinant α-syn aggregates induce different spreading processes and pathological alterations from the periphery to the CNS, which implies the possible source of heterogeneity among synucleinopathies.

## Materials and methods

### Preparation of α-syn fibrils

α-Syn monomers were produced as previously described [42]; the monomers had been purified and characterized. We used these monomers to generate strains B and C under the conditions shown in Table 1. The monomers were incubated for 7 days at a concentration of 0.5 μg/μl in different buffers (Table 1) at 37 °C with agitation speed of 1000 rpm, using 3-mm magnets in “Low Binding” 1.5-ml Eppendorf tubes. Fibrillation of the monomeric α-syn was confirmed with Thioflavin T binding assay and transmission election microscopy (TEM). Strain A was produced with protocols described previously [35]. Briefly, the monomeric α-syn was incubated in buffer (containing 50 mM Tris, 150 mM KCl, pH 7.5) at 37 °C and agitated in an Eppendorf Thermomixer at 600 rpm at 37 °C for 7 days. The α-syn PFFs were stored at − 80 °C until use. All strains were diluted in sterile phosphate-buffered saline (PBS) to 1 mg/ml and sonicated with a water-bath sonicator for 30 min before use.

**Table 1:** Production conditions of α-syn strains

Number	Buffer content	Buffer (pH)	Temperature (°C)	Duration (Days)	Agitation speed (rpm)	α-Syn Type
Strain A	50 mM Tris, 150 mM KCl	7.5	37	7	600	Full-length
Strain B	10 mM Tris, 50 mM NaCl	7.6	37	7	1000	Full-length
Strain C	10 mM MEZ	5.5	37	7	1000	Full-length

### Electron microscopy

The different strains of fibrils were thawed, filtered using 100 kD columns (Millipore), and reconstituted. Uranyl acetate was used for negative staining. Proteins were loaded onto the carbon-coated grids and imaged by a transmission election microscope (FEI Tecnai BioTwin 120 kV).

### Proteolytic digestion

The α-syn strains (0.5 mg/ml) were treated at 37 °C with proteinase K (3.8 μg/ml) (ThermoFischer Scientific, Waltham, MA), and aliquots were transferred at different time intervals following addition of phenylmethylsulfonyl fluoride (final concentration 1 mM) into preheated Eppendorf tubes maintained at 90 °C containing the loading buffer to arrest the cleavage reaction. After incubation at 90 °C for 10 min, the samples with different time courses were loaded to 15% SDS-PAGE followed by Coomassie brilliant blue staining.

### α-Syn aggregation assay in cultured cells

HEK293 cell line stably expressing A53T-mutant human α-syn with a green fluorescent protein (GFP) tag was generated in our lab. The cells were cultured in Dulbecco’s Modified Eagle Medium (DMEM) containing 10% (*v*/*v*) fetal bovine serum and 1% penicillin–streptomycin, and maintained at 37 °C in a 5% CO_2_ humidified environment. For fibril transduction, cells were plated at a density of 90,000 cells/cm^2^ on collagen G-coated 96-well plates, and incubated with equal volumes of Lipofectamine-2000 (ThermoFischer Scientific) final concentration 1.25% *v*/*v*) and α-syn strains (final concentration 1 μg/ml), both diluted in the OptiMEM medium, for 20 min for liposome formation. The cells were further incubated for 24 h after transduction. Then they were fixed with 4% paraformaldehyde (PFA) at room temperature (RT) for 15 min, washed 3 times with PBS, followed by immunohistochemistry with anti-pSer129 antibody and Cy3 goat-anti rabbit secondary antibody (Table 2). The slides were viewed and images were acquired using × 20 and × 40 objectives on a Zeiss Vert.A1 microscope. For each replicate, three random fields were chosen to measure the GFP density with image J and the number of cells exhibiting aggregates was counted. The numbers were summed and transformed to the percentage of different aggregate forms to the total number of aggregates. Colocalization of pSer129 and aggregates with GFP signals was measured by Pearson’s correlation coefficient (PCC) using image J plugin Coloc 2. Three independent replicates were analyzed. One-way ANOVA with Tukey’s multiple comparison post-hoc tests was performed to analyze the immunofluorescence intensity, co-localization and types of aggregation on GraphPad Prism 6.0.

**Table 2:** Primary and secondary antibodies in immunostaining

	Source	Vender	Catalog No.	Dilution	Application	Type
anti-pSer129	Rabbit	Abcam	ab51253	1:10,000	Immunohistochemistry	1st antibody
				1:1000	Immunofluorescence	
anti-Iba1	Rabbit	Wako	019–19,741	1:500	Immunohistochemistry	1st antibody
anti-Iba1	Mouse	Millipore	MABN92	1:1000	Immunofluorescence	1st antibody
anti-ChAT	Goat	Millipore	AB144P	1:100	Immunohistochemistry	1st antibody
Biotinylated anti-rabbit	Goat	Vector Laboratories	BA-1000	1:500	Immunohistochemistry	2nd antibody
Biotinylated anti-goat	Horse	Vector Laboratories	BA-9500	1:500	Immunohistochemistry	2nd antibody
Cy3 anti-rabbit	Goat	Jackson Immuno Research	139,290	1:500	Immunofluorescence	2nd antibody
Cy3 anti-mouse	Donkey	Jackson Immuno Research	715–165-150	1:200	Immunofluorescence	2nd antibody
Alexa Fluor® 647 anti-rabbit	Donkey	Jackson Immuno Research	711–605-152	1:200	Immunofluorescence	2nd antibody

### Cell viability assay

Cell viability was determined with the cell counting kit-8 (CCK-8) assay. Briefly, 100 μl of DMEM containing 10 μl CCK-8 was added to each well at the end of treatment and incubated in the 5% CO_2_ incubator at 37 °C for 2 h. The absorbance at 490 nm was measured with a microplate reader. The experiment was repeated for three times with three replicates in each treatment group. The cell viability was analyzed with one-way ANOVA followed by Tukey’s multiple comparison tests on GraphPad Prism 6.0.

### Mouse strains and procedures

Mice were housed in cages in groups of 3–4 per cage, with free access to food and water under a 12 h light/12 h dark cycle. All animal experimental procedures were performed following the specifications set by the Ethical Committee for Use of Laboratory Animals at Lund University, Sweden and at Northeastern University, China. Homozygous BAC-α-syn-GFP transgenic mice [43] were generated by inserting the human wild-type α-syn cDNA together with GFP into the fertilized eggs of C57BL/6 mice, under the control of the mouse α-syn promoter. The BAC-α-syn-GFP and C57BL/6 mice were injected with Strain A (10 μg), Strain B (10 μg), Strain C (10 μg), monomer (10 μg) or PBS (10 μl) at the biceps femoris unilaterally, and sacrificed at 180 dpi (*n* = 5 animals in all groups, except for *n* = 6 in the C57BL/6 with Strain A injection group).

### Intramuscular injection

Six-month-old BAC-α-syn-GFP transgenic mice and C57/BL6 wild-type mice were anesthetized with 50 mg/kg sodium pentobarbital intraperitoneal injection, shaved around the injection side, and unilaterally injected with 10 μg of α-syn or 10 μl of PBS by using a 10-μL Hamilton syringe. The needle was inserted 1 mm deep into the right biceps femoris muscle. Different syringes were utilized for each type of inoculum to prevent protein or sample mixture. The mice were placed on a warm pad for recovery and then returned to their home cages after injection.

### Behavioral tests

#### Footsteps assay

The behavioral tests were performed every 15 days from 45 dpi until sacrifice. The footsteps assay was performed according to the previous descriptions with minor modifications [44]. All mice were painted with non-toxic blue and red ink on the hind paws. A fresh sheet of filter paper was placed on the floor of the runway. In all cases, the first two and the last two steps in the testing apparatus were not used for analysis. The footprint patterns were collected and analyzed by measuring the vertical step length (stride), which is the distance travelled through the runway divided by the number of steps, and the step width (base), which is the distance between the right and left steps.

#### Inverted screen test [45]

A 43-cm long square-shaped mesh screen was prepared. The screen was wired with 1-mm diameter metal strings to form a net composed of 12-mm long squares. The screen was then surrounded by a 4-cm deep beading to prevent escaping. The mouse was placed on the center of the screen, and the screen was gently rotated 180° above a cushion. The behavioral response was scored based on the time of hanging: 1 = fell off between 0 s and 10 s, 2 = fell off between 10 s and 25 s, 3 = fell off between 25 s and 60 s, and 4 = did not fall off within 60 s.

### Tissue preparation

The mice (*n* = 3 in each group) were deeply anesthetized with sodium pentobarbital and then perfused intracardially with 0.9% sodium chloride (NaCl) followed by 4% PFA. The brain and spinal cord were post-fixed with 4% PFA at 4 °C overnight, and then immersed in 30% sucrose in 0.1 M PBS. The brain was sectioned with a frozen microtome (Leica, SM2010R, Germany) into 30-μm free-floating coronal sections and the spinal cord was sectioned into 30-μm free-floating horizontal sections. For muscle histology, the biceps femoris muscles were taken as whole-mount and were placed into ice-cold PBS to remove connective tissue. Then the muscles were fixed with 4% PFA in 0.1 M PBS, rinsed with PBS and stored in PBS at 4 °C until use.

### Immunohistochemistry

For immunostaining, the free-floating sections were antigen-retrieved in 0.1 M citrate acid buffer (pH = 6) at 80 °C for 40 min. The endogenous peroxidase was quenched by 3% hydrogen peroxide in 0.1 M PBS for 15 min at RT. The sections were pre-incubated with the blocking buffer (10% normal goat/horse serum and 0.3% Triton X-100 in 0.1 M PBS) at RT for 1 h, and incubated with anti-pSer129, anti-Iba1 or anti-ChAT (Table 2), followed by incubation with secondary biotinylated anti-rabbit antibody or anti-goat antibody (Table 2). Then the sections were incubated in standard ABC (Vector Laboratories, VEPK-6100) at RT for 1 h and treated with the DAB kit (Vector Laboratories, SK4100). The sections were dehydrated, and coverslipped with DPX for imaging with a microscope (Olympus BX53). For double immune-staining of Iba1 (a microglial marker) with phosphorylated α-syn, sections were pre-incubated with blocking buffer at RT for 1 h and incubated with primary antibodies anti-pSer129 α-syn and anti-Iba1, followed by incubation with secondary anti-rabbit Alexa Fluor 647 and anti-mouse Cy3 antibodies (Table 2). Images were acquired using a laser confocal microscope (Leica TCS SP8) with a sequential scanning mode. pSer129 α-syn intensity was quantified with ImageJ.

### Quantification of the staining and statistical analysis

Data of behavioral tests were analyzed by two-way ANOVA with Dunnett’s multiple comparison tests on GraphPad Prism 6.0. One-way analysis of variance (One-way ANOVA) tests were performed to statistically analyze the intensity of pSer129 α-syn immunohistochemistry in the spinal cord and brain regions and cell numbers of microglia and motor neurons were analyzed with one-way ANOVA followed by Tukey’s multiple comparison tests on GraphPad Prism 6.0. Comparison of pSer129 α-syn intensity between the ipsilateral and contralateral sides in the ventral horn of the spinal cord was performed with unpaired *t*-test.

### Muscle histology

For muscle histology, whole mounts of biceps femoris muscles were incubated for 2 h with 5 μg/ml Alexa-594-conjugated α-bungarotoxin (α-BTX) (Invitrogen, B13423). Images were taken with a laser confocal microscope (Leica TCS SP8). Quantification of motor endplate with morphological alterations was performed by counting more than 100 of motor endplates from three animals in each group.

## Results

### Characterization of different α-syn fibrils

**Fig. 1:**
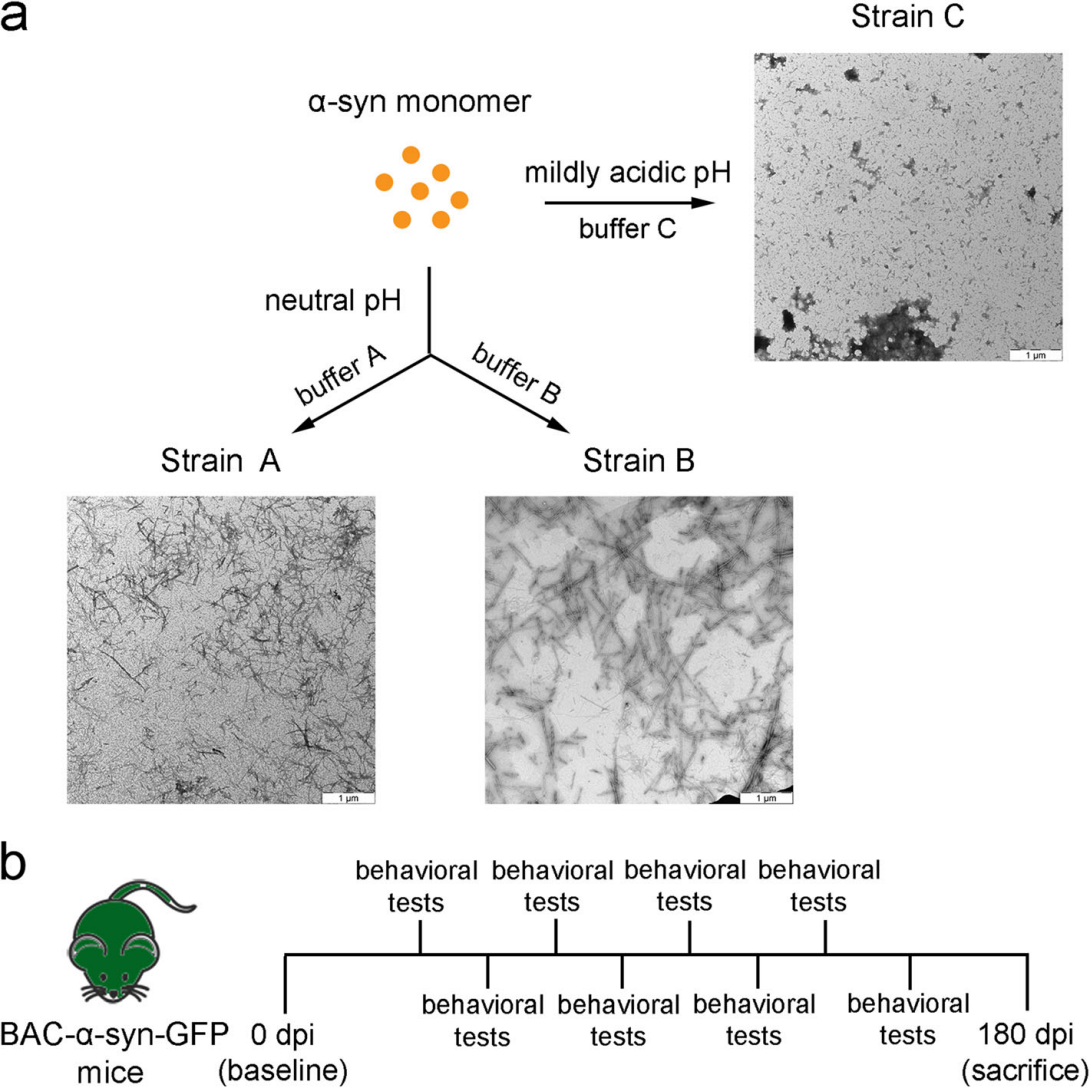
Generation of three strains under different conditions and schematic illustration of the experimental timeline. **a** Negatively stained TEM images of the fibril assemblies generated in different conditions. Scale bar, 1 μm. Buffer A: 50 mM Tris, 150 mM KCl, pH 7.5; buffer B: 10 mM Tris, 50 mM NaCl pH 7.6; buffer C:10 mM MEZ, pH 5.5. **b** Schematic of the timeline of the study

TEM of α-syn fibrillar polymorphs generated in three different conditions revealed that Strain A generated in buffer A (50 mM Tris, 150 mM KCl, pH 7.5) displayed a cylindrical structure and was short in length (Fig. 1a). Strain B assembled in lower ionic condition (10 mM Tris, 50 mM NaCl) and neutral pH was longer and less clumped than Strain A (Fig. 1a). A mildly acidic pH led to the generation of amorphous fibrils (Strain C) compared to the former two (Fig. 1a). To further compare the different fibrillar polymorphs, we assessed their proteinase K digestion profile on 15% SDS-PAGE. The proteinase K digestion of the three strains resulted in different digestion profiles, indicating their distinct structural characteristics (Fig. S1).

Next, we characterized the strains on a cultured cell line that stably expressed A53T-mutant human α-syn with a GFP tag, after cell liposome-mediated transduction (Fig. S2). In contrast to the cells transduced with α-syn monomers or PBS that only showed diffuse green cytoplasmic fluorescence, the cells exposed to Strain A, Strain B or Strain C displayed increased density and different morphologies of aggregates, which indicated the formation of α-syn (A53T)-GFP aggregates. In particular, the cells transduced with Strain B showed higher fluorescence intensity compared to the others (Fig. S2a, b). Importantly, PCC analysis revealed that in the cells transduced with Strains B and C, the aggregation was colocalized with pSer129 α-syn (*R* = 0.43 ± 0.1; *R* = 0.43 ± 0.02, respectively), and the positive correlation was lower in cells transduced with Strain A (*R* = 0.26 ± 0.02) (Fig. S2d). Also, there was phosphorylated α-syn formed after addition that was not aggregated (Fig. S2a, yellow arrow). Interestingly, there were differences in the morphology of aggregates among the strains. Strain A transduction resulted in 58.6% of small globular aggregates, which were slightly phosphorylated (Fig. S2a_3_, white arrow, Fig. S2e). The cells transduced with Strain B developed larger globular aggregates while the cells treated with Strain C developed a larger percentage (89.4%) of thread-like aggregates (Fig. S2a, arrowhead) on the border of the cells.

To compare the potential cytotoxicity of these strainsin vitro, we performed CCK-8 experiments. The results showed that when using lipofectamine for transduction, exposure to α-syn or saline did not cause a significant difference in cell viability (Fig. S2c).

### α-Syn-injected mice did not develop severe motor deficits after injection

**Fig. 2:**
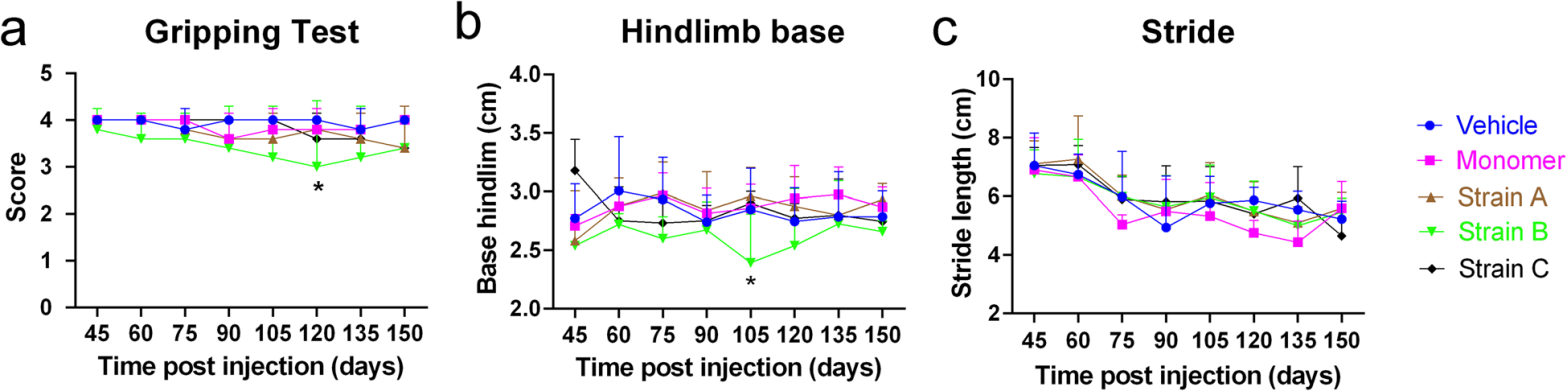
Motor deficits of the BAC-α-syn-GFP mice after intramuscular (IM) injection. **a** Score of gripping tests (two-way ANOVA with Dunnett’s multiple comparison tests, *n* = 5, *P = 0.0138). **b**, **c** Footprint assay. The hindlimb base after IM injection of Strain B was significantly shorter than the control at 105 dpi (two-way ANOVA with Dunnett’s multiple comparison tests, *n* = 5, *P = 0.0240), followed by a trend of recovery. No significant difference was found in stride. Mean ± SD

After validation of the α-syn fibrils, we performed the unilateral injection of the α-syn fibril strains into the mouse skeletal muscle (Fig. 1b), and assessed their motor alterations. The behavioral tests were performed every 2 weeks from 45 dpi until sacrifice. We observed a significant difference in the gripping ability in the inverted screen test and the hindlimb base in the footstep assay after IM injection of Strain B at 120 dpi and 105 dpi, respectively (Fig. 2a, b). There was a tendency of recovery for the gripping ability and the hindlimb base especially for the Strain B. In contrast, the mice injected with Strain C showed a steady slight decline in gripping test and stride in footstep assay with no significant difference (Fig. 2).

### α-Syn strains triggered propagation of pathology in the spinal cord and brain regions

**Fig. 3:**
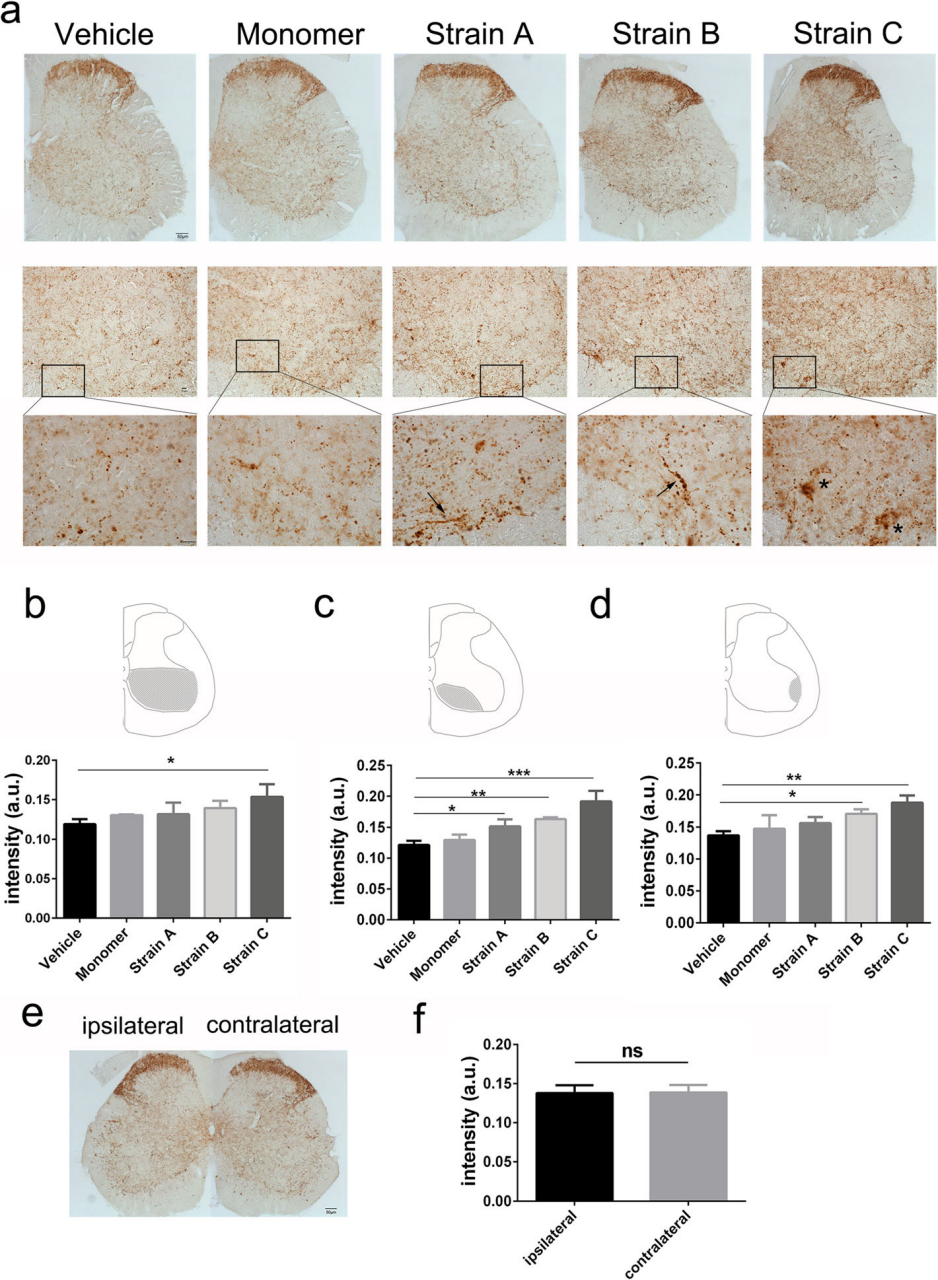
Distribution of phosphorylated α-syn in the lumbar spinal cord after injection of different α-syn strains. **a** Immunohistochemistry for pSer129 α-syn demonstrated α-syn pathology in the spinal cord among different groups. High magnification showed that pSer129 α-syn in motor neurons was mainly located in the lumbar spinal cord. α-Syn aggregation was present in neurites (arrows) and in cell bodies (asterisks). Scale bar, 10 μm. **b**–**d** Semi-quantitative analyses of pSer129 α-syn intensity (immunohistochemical staining) in the whole ventral part and areas of the lumbar spinal cord where motor neurons are mainly located (one-way ANOVA with Tukey’s multiple comparison tests, *n* = 3, *P < 0.05, **P < 0.01, ***P < 0.001). **e** Comparison between the ipsilateral and contralateral sides in the lumbar spinal cord in Strain B group. Scale bar, 50 μm. **f** Statistical analysis by unpaired *t*-test (*n* = 3, *P* > 0.05). Mean ± SD

Phosphorylation of α-syn is a common feature in PD pathogenesis, as over 90% of LBs are positive for phosphorylated α-syn [46, 47]. Therefore, we employed phosphorylated α-syn as a marker for α-syn inclusion, and used an antibody specifically against the serine 129 residue of α-syn. To investigate the seeding effects of different α-syn strains, we examined pSer129 α-syn pathology in the lumbar segment of the spinal cord, where the motor neurons innervating the hindlimb muscles reside. All the three strains managed to seed in the lumbar spinal cord after IM injection. As α-syn accumulation and aggregation increased with age in this mouse model [43], we also examined the endogenous α-syn in the vehicle and α-syn monomer groups. Compared to the control groups, the number of large puncta and pearl-like patterns which were positive for GFP and pSer129 α-syn increased (Fig. S4, arrow). Interestingly, the “original” α-syn shown by GFP was not completely phosphorylated (Fig. S4, arrowhead). Similar to the transgenic mice at old age, the inclusions were mainly in the neurites. LN-like profiles were present in the ventral horn of the spinal cord, especially in the neuropil around motor neurons in the three strain groups (Fig. 3a, arrows). The Strain C group also showed more aggregations inside the cell bodies (Fig. 3a, asterisks).

To further compare the seeding efficiency among groups, we quantified the positive signals in the whole ventral horn of the spinal cord (Fig. 3b–d). We found that the density in the ventral horn in Strain C group was significantly increased compared to the vehicle group (Fig. 3b). We also assessed the density in the sub-regions around the motor neurons and found that the Strain B and Strain C groups exhibited significantly higher signals than the control (Fig. 3c, d), while the Strain A group showed milder α-syn aggregation only in one motor neuron area (Fig. 3c). In addition, we compared the ipsilateral and contralateral sides in the lumbar spinal cord with Strain B injection, but no difference was found in signal intensity (Fig. 3e, f).

In contrast, we did not observe positive structures in the spinal cord in the wild-type mice after IM injection of Strain B (Fig. S3g), indicating that the seeding effect of the fibril strains required participation and expression of endogenous α-syn.

**Fig. 4:**
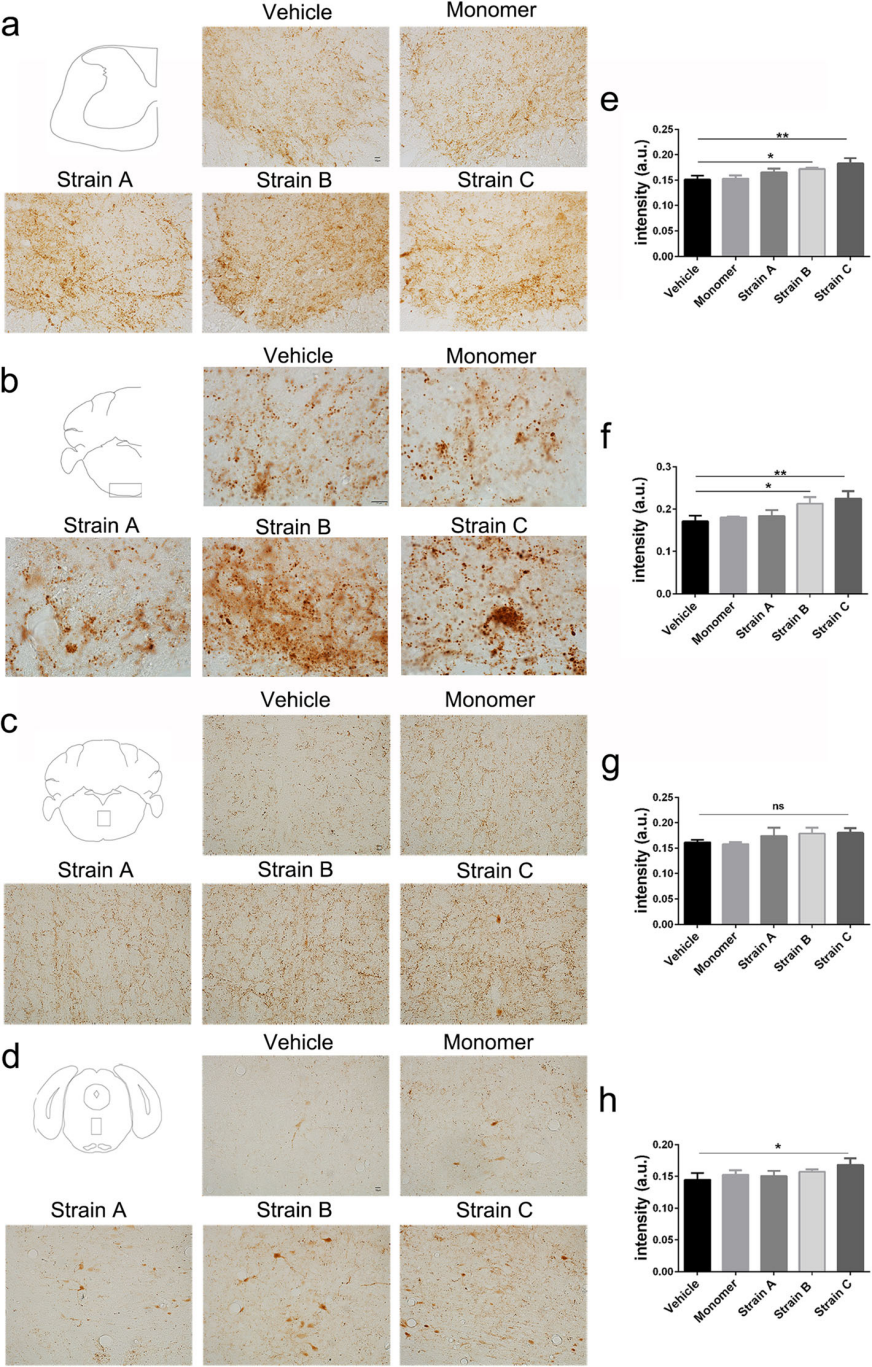
Propagation of α-syn pathology from spinal cord to brain in each group. **a–d** Distribution of pSer129 α-syn in the cervical spinal cord and propagation of α-syn pathology from skeletal muscle to the upper brainstem including medulla oblongata and pons of the brain. Scale bar, 10 μm. **e–h** Statistical analysis in the above brain regions (one-way ANOVA with Tukey’s multiple comparison tests, *n* = 3 *P < 0.05, **P < 0.01). Mean ± SD

To compare the propagation efficiency and inoculated aggregation of α-syn pathology of the three strains after IM injection, we performed immunostaining with anti-pSer129 antibody in the cervical spinal cord and the brain. We found that the pSer129 α-syn-positive inclusions in the cervical spinal cord increased after IM injection of the strains compared to the controls (Fig. 4a–d). In addition, the pathological patterns differed among different strains. Strain C induced efficient inclusion propagation to the upper brain regions and caused the most severe α-syn pathology in the corticospinal tract in the medulla oblongata, in which larger inclusions were seen (Fig. 4b). Strain B induced more LN-like pathology in this region (Fig. 4b). There were more LN-like structures and cell body staining around the median of the upper parts of the brainstem in the Strain B and Strain C groups, while Strain A caused mild pathology in the brain regions (Fig. 4c, d). Endogenous α-syn pathology was present in the substantia nigra, ventral tegmental area, hypothalamus and motor cortex (Fig. S3a–c) at 12 months of age, with no difference among the groups (Fig. S3d–f).

### Degeneration of motor neurons and neuromuscular junctions

**Fig. 5:**
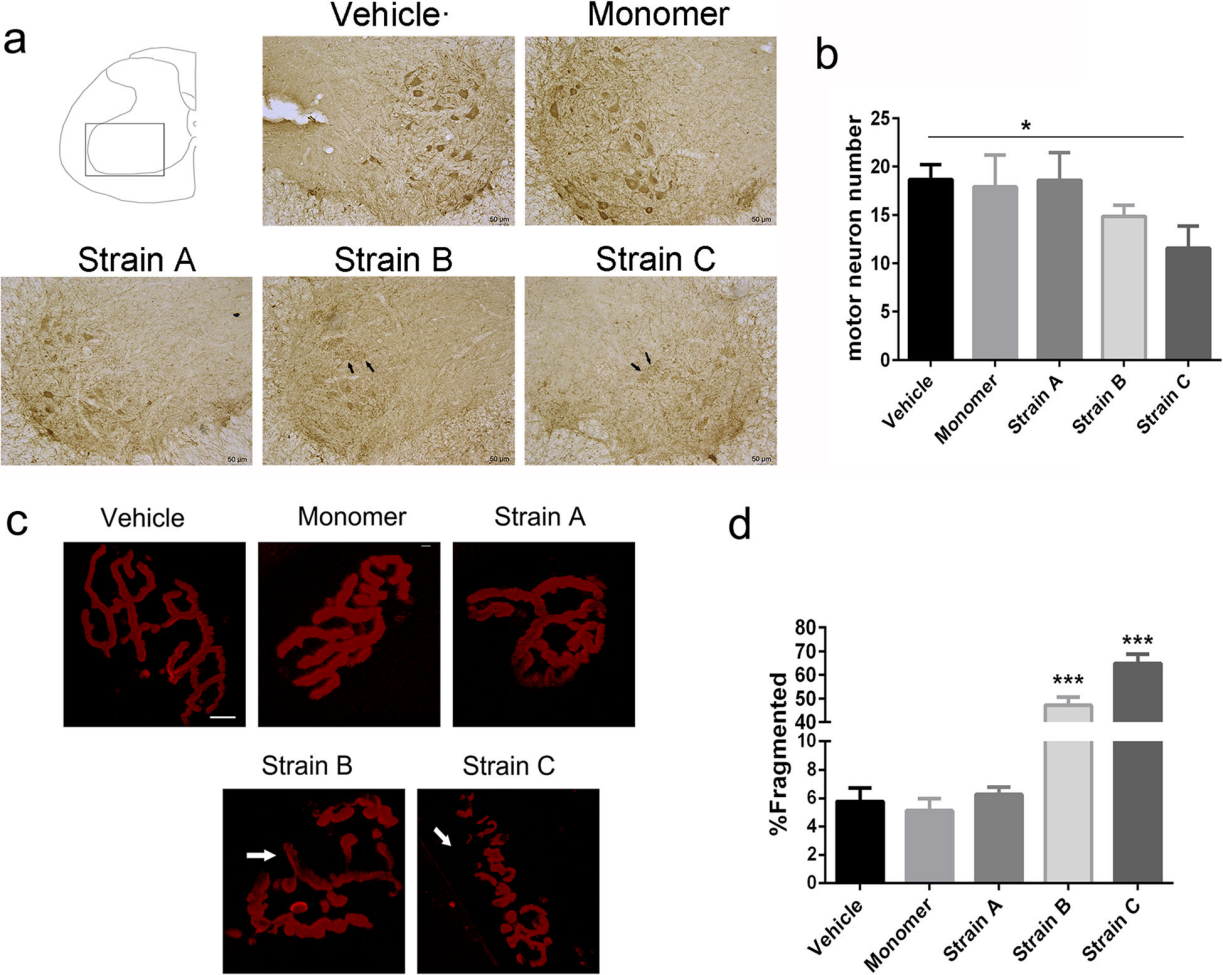
Motor neuron degeneration in the spinal cord and neuromuscular junction (NMJ) neurodegeneration in the skeletal muscle after IM injection of α-syn strains in the BAC-α-syn-GFP mice. **a** Immunohistochemistry analysis of ChAT demonstrated distribution of motor neurons in the spinal cord after injection. Scale bar, 50 μm. **b** Numbers of motor neurons marked by ChAT in the ventral horn of the lumbar spinal cord post injection in different groups. One-way ANOVA, *n* = 3, *P = 0.0175 with Tukey’s multiple comparison tests, mean ± SD. **c** Morphological changes of motor endplates in Strain B and Strain C groups. AChRs were labeled with Alexa-594-α-BTX (red) in biceps femoris. The NMJs became fragmented in the Strain B and Strain C groups, and some islands of NMJ showed lack of AChRs (arrows) Scale bar, 10 μm. **d** Quantification of frequency of abnormalities in NMJs from biceps femoris. Mean ± SD from at least 100 NMJs from 3 mice

To detect the cell toxicity caused by α-syn spread and seeding and how these strains influence motor neurons in the spinal cord, we examined motor neuron degeneration in the ventral spinal cord using an antibody for choline acetyltransferase (ChAT), which is a key enzyme for acetylcholine synthesis as a cholinergic motor neuron marker. We found that the total number of motor neurons in the ventral horn significantly decreased after IM injection of Strain C compared to the vehicle group (Fig. 5a, b), indicating significant cytotoxicity. In addition, some neurons in the spinal cord in this group showed unclear boundaries and were lightly stained under the same staining conditions (Fig. 5a, arrows), suggesting a degeneration process with reduced ChAT content. The Strain B group also showed a trend of neuronal degeneration but with no statistical difference (Fig. 5b). In comparison, neurons in the vehicle and monomer groups were robustly stained for ChAT (Fig. 5a, b).

Neuromuscular junction (NMJ) fragmentation has been found in muscles of old mice compared with young mice [48], suggesting the occurrence of synaptic alterations with aging. Motor endplates were labeled with Alexa-594-tagged α-BTX, a highly selective marker for acetylcholine receptors (AChRs). We assessed the morphology of NMJs in the skeletal muscle, which is innervated exclusively by a branch of the sciatic nerve. We found that 64.92% of motor endplates presented with fragments in the Strain C group and 47.19% of motor endplates displayed morphological changes in the Strain B group (Fig. 5c), while only 6.27% of motor endplates showed fragmentation in the Strain A group. In the α-syn monomer and vehicle groups, the percents were 5.13% and 5.78%, respectively (Fig. 5d). The degenerating motor endplates were fragmented into segments, and some showed weak fluorescence because of the loss of AChR (Fig. 5c, arrows).

### Inflammation in the spinal cord after injection of different α-syn strains

**Fig. 6:**
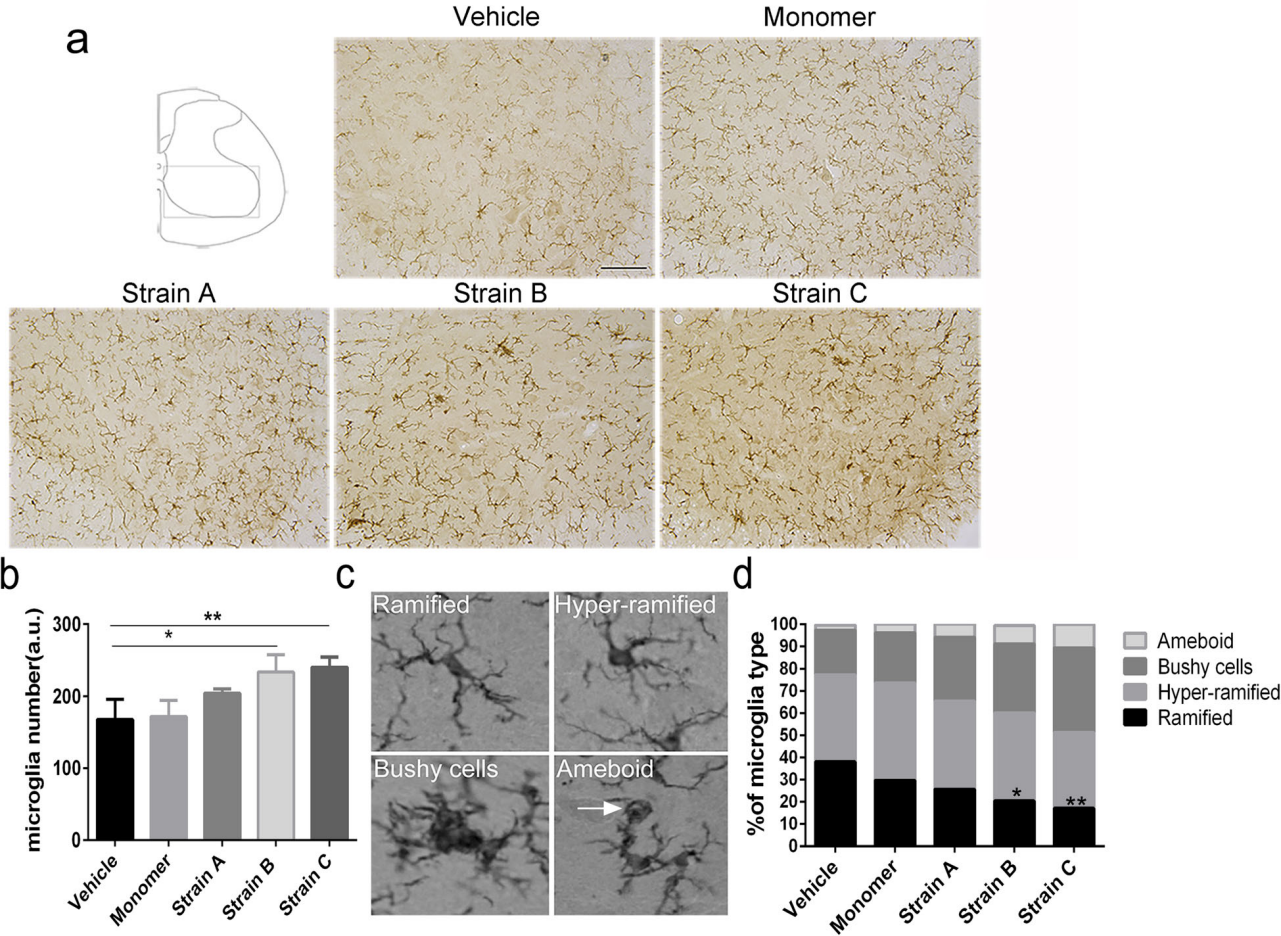
Microglia-associated neuroinflammation in the spinal cord in BAC-α-syn-GFP mice. **a** Immunostaining of Iba1 showed the distribution of microglia in the lumbar spinal cord. Scale bar, 100 μm. **b** Numbers of microglia in the ventral horn of the lumbar spinal cord after injection of different α-syn fibrils. One-way ANOVA, *n* = 3, *P < 0.05, **P = 0.0034 with Tukey’s multiple comparison tests. **c** Morphology of Iba1-positive cells revealed ramified (surveillance microglia), hyper-ramified (intermediate) microglia, and un-ramified (bushy or amoeboid) microglia. **d** Percentage of Iba1-positive cells according to the morphology in the lumbar spinal cord after IM injection in each group (*n* = 3, *P < 0.05, **P < 0.01). The total numbers of the microglia at stages 2–4 increased in Strain B and Strain C groups (*n* = 3, *P < 0.05), analysed by one-way ANOVA with Tukey’s multiple comparison tests. Each bar shows mean ± SD

Neuroinflammation has been reported to be involved in PD pathogenesis. The activation of microglia is thought to cause the loss of dopaminergic neurons in PD [49, 50]. We therefore detected whether neuroinflammation was present in the PNS-to-CNS propagation model. To test this, microglia were stained with the anti-Iba1 antibody. The total number of microglia in the ventral horn significantly increased in the Strain B and Strain C groups with varied degrees of activation (Fig. 6a, b). The microglia were classified into four stages according to the morphological transformation [51]: ramified (resting), hyper-ramified (reactive or intermediate), bushy and amoeboid (Fig. 6c). After IM injections of Strains B and C, the percentage of un-ramified (bushy or amoeboid) microglial cells in the ventral horn increased considerably, and the ramified microglia decreased accordingly (Fig. 6d). Moreover, microglia and pSer129 α-syn were not co-localized in the lumbar spinal cord (Fig. S5).

## Discussion

In the current study, we investigated the effects on α-syn seeding and propagation induced by three α-syn strains injected in the periphery in transgenic mice that express human wild-type α-syn. The seeding and propagation efficiency exhibited a strain-dependent pattern, in which one strain generated in mildly acidic buffer condition showed the most effective seeding and propagation from the periphery to the CNS.

### Comparison of morphology and biochemical characteristics of α-syn PFFs generated under different conditions

It is widely recognized that the fibrilization of α-syn is a process of two steps including nucleation and elongation. The process begins with a lag phase reflecting nucleation and further growth and reaches a fibrillary steady phase [52, 53]. The structural polymorphism is mainly due to the different folding of the β-sheet and the interactions between the amino-acid side chain and the environment. Our results showed that the strain generated in mildly acidic pH displayed most efficient seeding and propagation, indicating that pH is an important factor that affects the formation of fibrils. In a previous study, reduction of pH from 7 to 4 resulted in an amorphous appearance rather than a fibrillar structure of aggregates, without preventing the formation of Thioflavin T-binding cross-β amyloid structure [54]. In addition, lower pH can affect the lag time and the elongation rate, leading to acceleration of the kinetics of α-syn fibrillation [55]. Buell et al. have found that the secondary nucleation is accelerated at mildly acidic pH by exposure to hydrophobic cores [56]. These results suggest that pH can affect the nucleation and fibril elongation into a specific structure, and further induces different levels of α-syn pathology in vivo.

The salt concentration in buffer is another important condition for the generation of the strains. In this study, strain B, which was generated at neutral pH but a slightly lower salt concentration compared to the strain A, was more effective than Strain A in both seeding and propagation. At pH 7.0, increasing the salt concentration can result in a shorter lag time and an increased growth rate [54]. Peelaerts et al. have shown that α-syn strains assembled from the same precursor α-syn but in different buffers have varied biological activities and abilities of transmission in vivo [35, 36]. More recently, Lau et al. generated two types of strains with salt (S) and without salt (NS), respectively [40]. They found that the S fibrils were structurally shorter and fragmented at a higher rate than the NS fibrils, and these two fibrils promoted pSer129 pathology in different brain regions. Similarly, Suzuki et al. have also illustrated that the α-syn strains produced without salt have higher prion-like seeding activity than those assembled in the presence of salt in the mouse brain [57].

The aggregation propensity of α-syn has been widely exploited to produce aggregated recombinant α-syn protein. In addition to the above mentioned factors, there are many more factors that can affect the biochemical, biophysical and even structural properties of α-syn fibrils, such as temperature [55, 58], α-syn mutants [59–62], presence or absence of agitation [58, 63, 64], agitation speed, duration time, and initial concentration of α-syn [58, 64, 65]. In addition, questions remain unclear regarding the of fibrils that reflect the de novo α-syn aggregates in cells and in the human body, and the extent to which these fibrils generated under in vitro conditions represent the physiological or pathological composition. Each type of fibrils may represent α-syn aggregates at a special setting, such as at different ages, in response to different aggregating triggers, in different genetic backgrounds, as a result of protein homeostasis, and even in the presence of different metal ion contents, or perhaps these fibrillar proteins are only chemically synthesized in vitro that simulate protein aggregation in vivo.

### Different backgrounds of transgenic mice play a role in seeding and transmission

In 2014, Sacino et al. first reported that intramuscular injection of α-syn PFFs induces CNS α-syn pathology along the neuroanatomical connectivity as well as a rapid motor phenotype in the M83 homozygous and heterozygous transgenic mice that express A53T α-syn [41]. In our model, the LN-like structures were detected in the lumbar spinal cord and the medulla oblongata with strain-specific pathology, indicating α-syn transmission in the neuronal network. We confirmed the induction of α-syn transmission to the CNS by IM injection of α-syn fibrils in the mouse model expressing human wild-type α-syn. According to our results, we speculate that the pathology may be affected by different background of a mouse model, such as the genetic background, the endogenous substrate, the type and resource of injected materials, and/or the type of receiving cells (i.e. different injection sites). The homozygous M83 mice develop intricate and severe motor impairments such as loss of weight, reduction of ambulation, hindlegs partial and then general paralysis at as early as 8 months of age [66]. In contrast, the model used in this study displays an age-dependent α-syn accumulation without severe behavioral alterations [43]. Also, for patients, symptoms usually develop when approximately 60% of the substantia nigra dopaminergic neurons are lost [67], indicating that neurodegeneration occurs earlier than clinical diagnosis of PD. Of note, for the M83 mouse line, expression of α-syn is regulated by the neuron-specific hamster prion protein gene promoter, leading to α-syn neuronal pathology and astrogliosis in the subcortical midbrain and brainstem which are related to motor pathways. In our model, the expression of human wild-type protein fused with GFP was driven by the mouse promoter, which caused slow and progressive α-syn accumulation and aggregation.

It remains unknown to which extent the endogenous α-syn in the α-syn over-expression background suppresses or enhances α-syn aggregation and propagation. In this study, our model exhibited a more prolonged and progressive pathology in relevant brain regions and the motor deficits appeared at a late stage, suggesting that overexpressing human wild-type α-syn could better mimic the patient condition [68, 69] than the A53T mutation, which only accounts for a small proportion of PD patients [70]. We have previously reported that our mouse model develops pathology in an age-dependent manner in the enteric neurons of the gastrointestinal tract of mice [71, 72]. This model, which develops symptomatic and pathological features of early PD, is suitable for studying peripheral pathology. We did not observe positive signals of pSer129 in the wild-type mice with all treatments, indicating that the high amounts of α-syn in vivo in the recipient animals play a role in exogenous α-syn propagation.

It has been reported that allelic interference in transgenic mice expressing both mouse and human prion protein (PrP) leads to the inability of propagation of human PrP strains [73, 74]. Accordingly, α-syn transmission studies have utilized mice, in which their endogenous α-syn was genetically knocked out, to avoid the transmission barrier from species between mouse and human. Interestingly, several studies injected extracts from MSA patients into mouse models expressing human wild-type or A53T/A30P mutant protein on a mouse knockout background [22, 75], and found that the MSA patient-sourced seeds support the prion-like propagation of pathological α-syn in the absence of endogenously expressed mouse α-syn, but without rapid disease onset or α-syn deposition, which indicated that the expression of mouse endogenous α-syn is not a crucial factor for transmission of α-syn in mice.

### Importance of the different injected materials and injection model divergence

Previously, it has been reported that in M83 homozygous and heterozygous mice, the disease process is significantly accelerated by intracerebral (IC) inoculations of brain homogenates from sick M83 mice, or of recombinant α-syn fibrils [4, 23, 29]. The MSA-inoculated mice developed disease onset at shorter incubation time (120 dpi). However, the M83 heterozygous mice injected with brain homogenate from PD or DLB patients displayed incubation times as long as 540 dpi. Some other studies also found that PFFs induced pathology at a range of time points. Remarkably, in the same model (M83 heterozygous mice), discrepancies in transmission and phenotype were observed upon the same IC injection, suggesting that injection of different materials can induce a strain-dependent pathology in vivo. Recent reports have revealed the presence of different α-syn strains in different synucleinopathies [38, 76–78], which are even able to be amplified in test tube conditions [38, 77, 78], providing new opportunities to study the α-syn strain-dependent properties of aggregation, propagation and cytotoxicity.

Peripheral administration including intramuscular [41], intraperitoneal [79], intraglossal [79], intravenous [80, 81] and even oral [81] infusions of α-syn PFFs has been performed in M83 mice and all leads to the development of α-syn pathology. We speculate that different injection sites require different fibril doses to induce pathology. Moreover, it has been found that α-syn pathology induced by IM injection with PFFs is largely dose-independent [82]. In another study, M83 heterozygous mice with IM injection of wild-type fibrils developed symptoms at 135 dpi [83]. Hence, it is possible that more severe α-syn pathology could be detected with prolonged incubation time with Strain A.

### Considerations for α-synuclein assembly features and quality control for research

Here we provided new evidence indicating that the process of aggregation is affected by buffer composition and other conditions, resulting in different extents of α-syn pathology in vivo. However, it is worth mentioning that the variations of assemblies between laboratories or batches are a factor for the variations in results [28, 30, 84, 85]. In this study, we used the same method (α-syn fibril sonication in a mild water bath sonicator for 30 min) as in a previous IM injection study [41]. It has been reported that the degree of sonication of α-syn fibrils can greatly influence the seeding effect of α-syn accumulation both in vitro and in vivo [58]. Volpicelli-Daley et al. also emphasized that it is crucial to utilize a sonicator with a probe for α-syn fibril sonication in their in vitro seeding study [86].

Another important point is that we did not take into account the possible effect of endotoxins, as α-syn is expressed in and purified from *E. coli*, which contains native endotoxins such as lipopolysaccharide (LPS). It has been reported that the IC or peripheral injection of LPS could induce α-syn pathology in homozygous M83 mice [87, 88], and LPS has been shown to cause α-syn accumulation, dopaminergic degeneration and motor dysfunction [30, 89, 90]. In addition, LPS is a potent activator of immune response. Rutherford et al. have reported that it is difficult to completely remove LPS from α-syn PFFs [91]. As for Lewy pathology, they found that IC injection of PFFs in hemizygous M83 mice in the presence or absence of LPS caused a difference in  inclusion distribution in only one brain region (cortex) [91]. Overall, endotoxin is a potential factor that affects α-syn pathology and inflammatory response, therefore should be controlled in PFFs injection models.

## Conclusions

In this study, we found that IM injection of α-syn strains did not induce severe motor phenotypes in a human wild-type α-syn transgenic mouse model. The α-syn strains generated by different protocols seeded and propagated differently and one strain generated in mildly acidic pH condition led to the most severe α-syn pathology and showed toxicity during retrograde axonal transport. Our work provides new evidence to support the idea that polymorphism of fibrillar α-syn could lead to different propagation patterns in α-syn pathology. Further studies need to be done to compare the fibrils generated in vitro with strains of pathological seeds found in patients with α-synucleinopathies.

## Supplementary Information

**Additional file 1: Figure S1.** The digestion of the strains by proteinase K. **Figure S2.** α-Syn aggregation assay showing differential seeding effects in cultured α-syn (A53T)-GFP cell line. **Figure S3.** Distribution of α-syn in the midbrain and forebrain of BAC-α-syn-GFP mice and spinal cord in wild type mice. **Figure S4.** Colocalization analysis of pSer129 α-syn and native α-syn-GFP in the lumbar spinal cord in BAC-α-syn-GFP mice. **Figure S5.** Colocalization analysis of pSer129 α-syn and microglia in the lumbar spinal cord in BAC-α-syn-GFP mice.

## Data Availability

The materials used and/or analyzed during the current study are available from the corresponding author on reasonable request.

## Abbreviations

AChR: Acetylcholine receptor
BAC: Bacterial artificial chromosome
CCK-8: Cell counting kit-8
ChAT: Choline acetyltransferase
CNS: Central nervous system
DLB: Dementia with Lewy bodies
dpi: Days post injection
GFP: Green fluorescent protein
IC: Intracerebral
IM: Intramuscular
LB: Lewy body
LN: Lewy neurite
LPS: Lipopolysaccharide
MSA: Multiple system atrophy
NMJ: Neuromuscular junction
PCC: Pearson’s correlation coefficient
PBS: Phosphate buffered saline
PD: Parkinson’s disease
PFA: Paraformaldehyde
PFF: Preformed fibril
PNS: Peripheral nervous system
PrP: Prion protein
TEM: Transmission electron microscopy
α-syn: Alpha-synuclein
α-BTX: α-Bungarotoxin
