# Supplementary material for: Differential seeding and propagating efficiency of α-synuclein strains generated in different conditions
Source: Transl Neurodegener. 2021 Jun 21;10:20. doi: 10.1186/s40035-021-00242-5 (PMC8215826; doi:10.1186/s40035-021-00242-5)
Supplement: Supplementary file 1 — Additional file 1: Figure S1. The digestion of the strains by proteinase K. Figure S2. α-Syn aggregation assay showing differential seeding effects in cultured α-syn (A53T)-GFP cell line. Figure S3. Distribution of α-syn in the midbrain and forebrain of BAC-α-syn-GFP mice and spinal cord in wild type mice. Figure S4. Colocalization analysis of pSer129 α-syn and native α-syn-GFP in the lumbar spinal cord in BAC-α-syn-GFP mice. Figure S5. Colocalization analysis of pSer129 α-syn and microglia in the lumbar spinal cord in BAC-α-syn-GFP mice. [file 40035_2021_242_MOESM1_ESM.pdf]

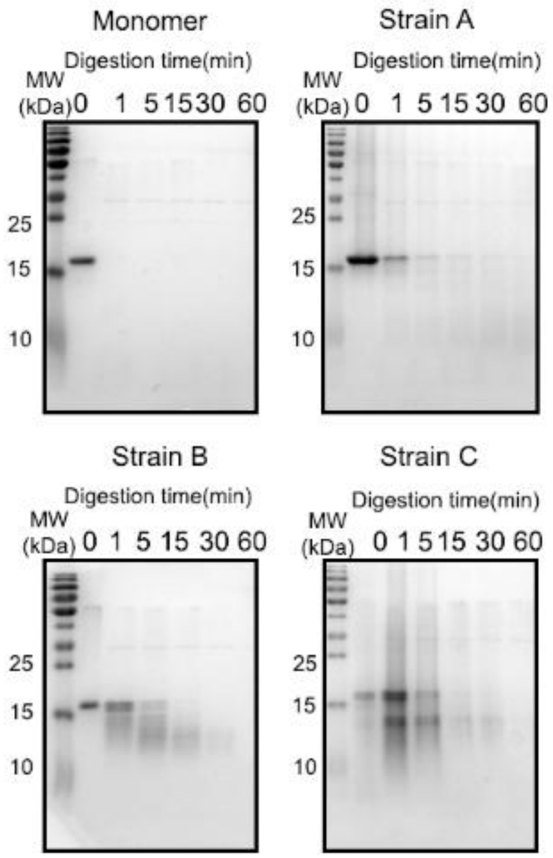

Figure S1. The digestion of the strains by proteinase K. Proteinase K was added and the samples were collected from 0 to 60 min. Each strain produces distinct banding profiles of insoluble α-syn species, and then assessed by 15% SDS–PAGE followed by Coomassie brilliant blue staining.

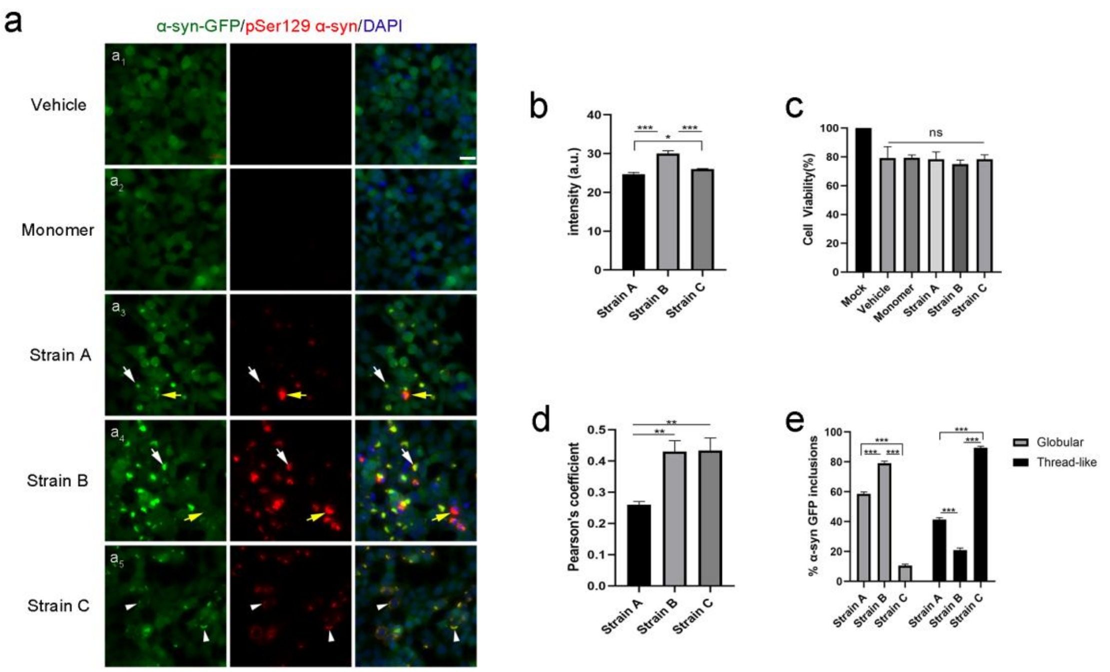

Figure S2. α-Syn aggregation assay showing differential seeding effects in cultured α-syn (A53T)-GFP cell line. **a** α-Syn-GFP and immunofluorescent staining with pSer129 α-syn (red). Scale bar = 50 μm. **b** α-Syn-GFP intensity after exposed with α-syn strains. Strain B caused the highest fluorescence intensity, which was significantly different from the other two, and the Strain C was more effective than the Strain A. Statistical analysis by One-way ANOVA (n = 3, * P <0.05 ** P <0.01). Bar shows mean ± SD. **c** Cell viability measured by CCK-8. The cells exposed to the three strains exhibited no significant difference compared to the vehicle group (n = 3). Bar shows mean ± SD. **d** PCC for colocalization of pSer129 α-syn and α-syn (A53T)-GFP aggregation. Statistical analysis by One-way ANOVA (n = 3, ** P <0.01). Bar charts show mean ± SD. **e** Percentage of aggregation patterns (globular and thread-like) caused by the three strains. Statistical analysis by one-way ANOVA (n = 3, *** P <0.001). Bar shows mean ± SD

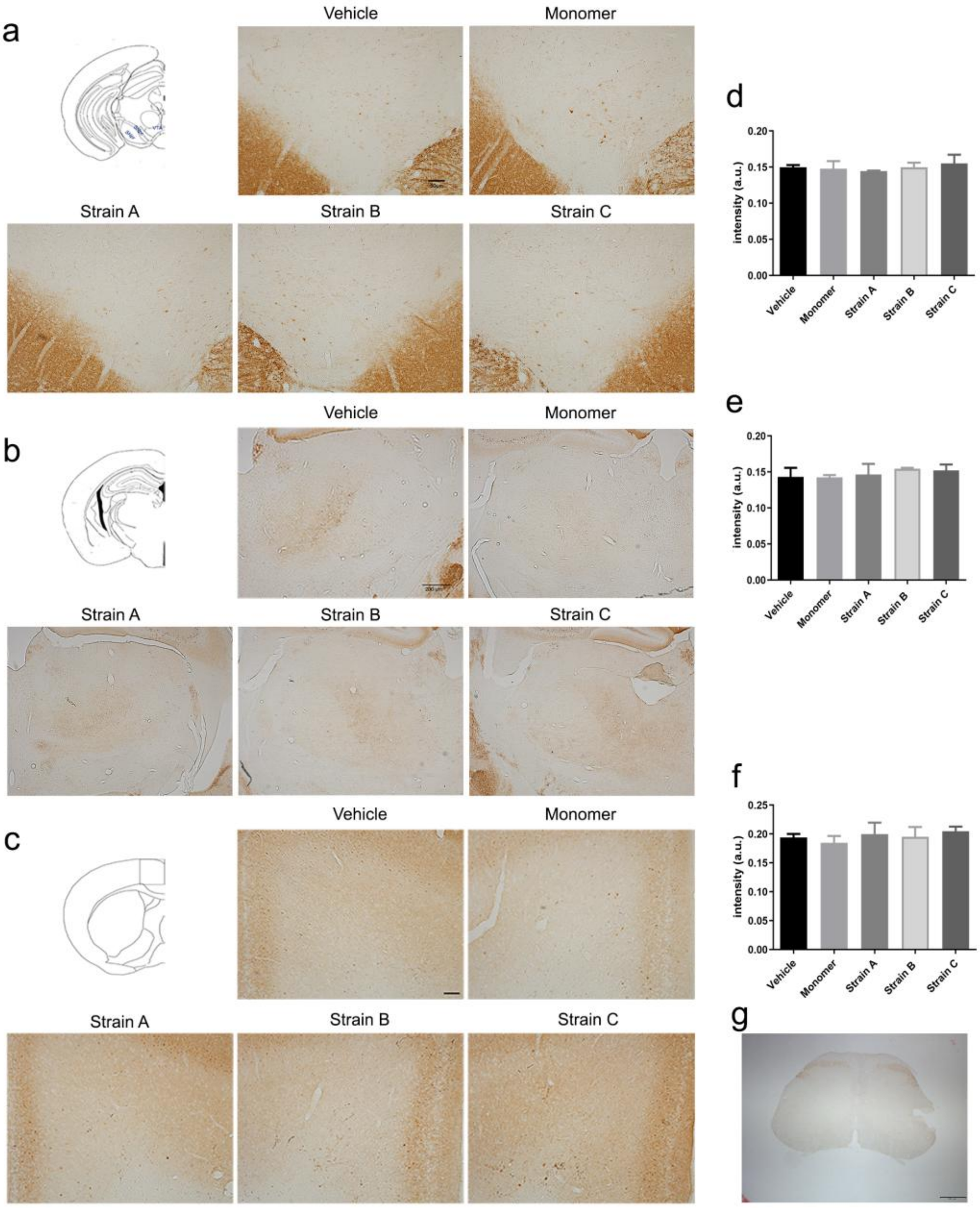

Figure S3. Distribution of α-syn in the midbrain and forebrain of in BAC-α-syn-GFP mice and spinal cord in wild type mice. **a-c** Distribution of pSer 129 α-syn in the upper brain regions. A similar amount of endogenous α-syn was accumulated in upper brain regions, the substantia nigra, thalamus, and the motor cortex, which did not increase significantly after IM injection. **d-f** Statistical analysis of brain regions by one-way ANOVA, n = 3, with Tukey's multiple comparison tests. Each bar shows mean ± SD. **g** pSer 129 α-syn staining in the lumbar spinal cord in wild type mice. No

accumulation of α-syn in the spinal cord in wild type mice injected with Strain B and histological analysis performed together with the BAC-α-syn-GFP mice. Scale bar A, C = 50 μm. Scale bar B, G = 200 μm.

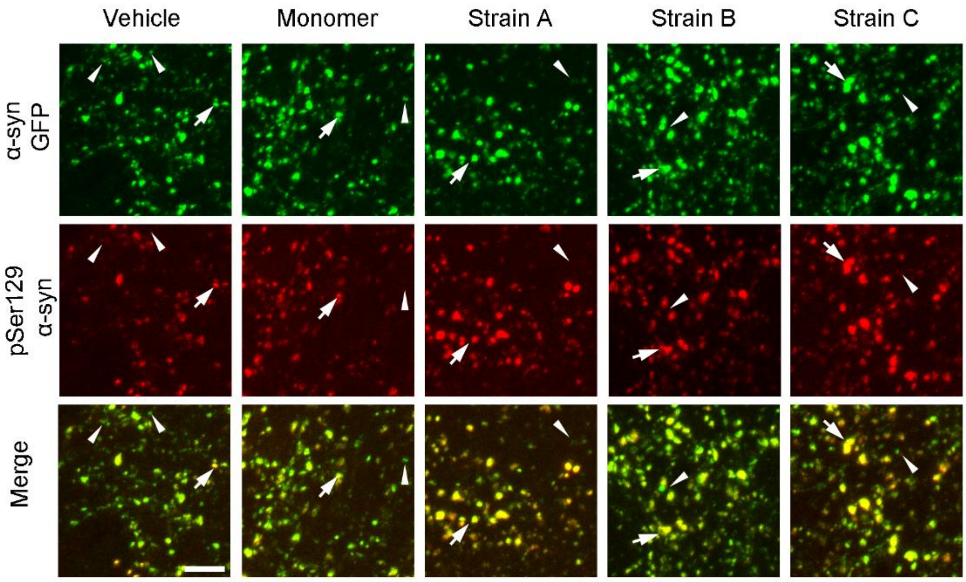

Figure S4. Colocalization analysis of pSer129 α-syn and native α-syn-GFP in the lumbar spinal cord in BAC-α-syn-GFP mice. The native and endogenous α-syn-GFP profiles(green) were mainly small granular and puncta in the lumbar spinal cord in the control group. After IM injections with α-syn strains (A, B, C), large puncta and pearl-like positive profiles increased, they are largely positive for GFP (native α-syn) and pSer129 α-syn (red) (arrows), some small puncta were only positive for GFP, but not for pSer129 α-syn (arrowheads). Scale bar = 10 μm.

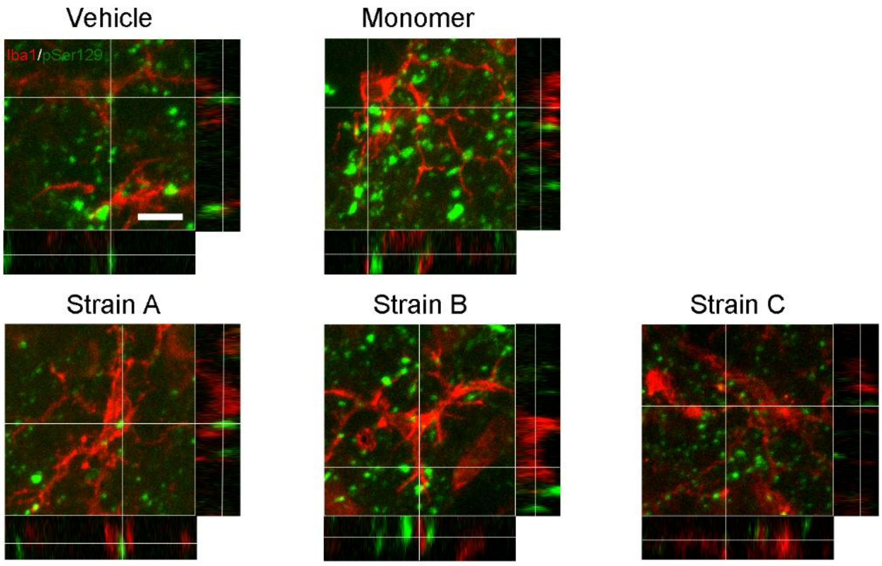

Figure S5. Colocalization analysis of pSer129 α-syn and microglia in the lumbar spinal cord in BAC-α-syn-GFP mice. Maximal projection of Z-stack confocal images, in which double labeling of pSer129 α-syn (green) and microglia (red) in each group showed pSer129 α-syn was not in microglia. Scale bar = 10 μm
